# The effects of strength and conditioning interventions on serve speed in tennis players: a systematic review and meta-analysis

**DOI:** 10.3389/fphys.2024.1469965

**Published:** 2025-01-07

**Authors:** Nuannuan Deng, Kim Geok Soh, Fan Xu, Xinggang Yang

**Affiliations:** ^1^ Department of Physical Education, School of General Education, Sichuan Fine Arts Institute, Chongqing, China; ^2^ Department of Sports Studies, Faculty of Educational Studies, Universiti Putra Malaysia, Selangor, Malaysia; ^3^ College of Physical Education, Chongqing University, Chongqing, China

**Keywords:** strength training, ball speed, rate of force development, performance, overhead sport

## Abstract

**Background:**

Tennis performance is highly influenced by serve speed. This review aimed to evaluate and quantitatively compare the efficacy of popular strength and conditioning (S&C) training methods in enhancing the speed of the ball in the serves of tennis players.

**Methods:**

Following PRISMA guidelines, a systematic search was conducted in the Scopus, Web of Science, SportsDiscuss, and PubMed databases without date constraints, up to July 2024. Studies included in this meta-analysis met PICOS criteria: a) randomized controlled trials with healthy tennis players, b) isolated or combined S&C training programs, c) evaluation of tennis serve speed, and d) adequate data to compute effect sizes (ESs). The PEDro scale was used to assess methodological quality.

**Results:**

Out of 271 identified papers, 16 studies of moderate to high quality were included in the meta-analysis. Resistance training demonstrated a small but significant effect on serve speed (ES = 0.53; *p* < 0.001), while multimodal training exhibited a moderate and significant effect (ES = 0.79; *p* = 0.001). However, core training did not have a significant effect on serve speed (ES = 0.32, *p* = 0.231).

**Conclusion:**

The findings suggested that S&C interventions, including resistance and multimodal training, were beneficial for increasing serve speed in tennis players. Further high-quality research is recommended to confirm this conclusion.

**Systematic Review Registration:**

https://www.crd.york.ac.uk/prospero/display_ record.php?RecordID=519790, identifier CRD42024519790.

## Introduction

The serve is one of the most important strokes in modern tennis due to its significant influence on match outcomes and frequent execution during gameplay (Colomar et al., 2022). It enables players to secure points through short rallies, with the percentage of points won after the first serve averaging around 72%–81% (Fett et al., 2021). Moreover, a successful first serve has become a powerful weapon for scoring directly or seizing immediate control of a rally (Klaus et al., 2017). In professional tennis, both men and women are capable of serving at speeds exceeding 200 km/h (Fett et al., 2020). Meanwhile, practitioners widely recognize that the effectiveness of a player’s serve largely depends on the speed at which the ball is hit (Landlinger et al., 2012; Baiget et al., 2023a). Studies have shown a strong correlation between serve speed, the ability to win match points (Fett et al., 2020), and ranking position (Ulbricht et al., 2016). Therefore, it is clear that generating high ball speed in the serve is essential for high level tennis performance (Whiteside et al., 2013). Consequently, players and coaches dedicate significant time to developing and refining training strategies to maximize serve speed without compromising accuracy (Fernandez-Fernandez et al., 2016).

Strength and conditioning (S&C) training is widely utilized to enhance sports performance. For example, Ehlert (2020) synthesized findings showing that S&C interventions led to average increases of 4%–6.4% in clubhead speed, ball speed, and distance measures in golf. Similarly, Ramachandran et al. (2022) reported a moderate and significant effect of plyometric training on bowling speed in cricket fast bowlers (standardized mean difference (SMD) = 0.75). Kwok et al. (2021) recommended incorporating S&C training into swimmers’ routines, citing its positive impact on swimming performance (SMD = −0.39 to 0.37). Genevois et al. (2013) also demonstrated an 11% increase in forehand drive ball speed in adult tennis players following S&C training. Meta-analyses by Deng et al. (2022), Deng et al. (2023) and Fleming et al. (2023) have highlighted the significant benefits of evidence-based training programs on tennis serve performance. Deng et al. (2022) found that plyometric training significantly enhanced maximal serve speed (effect size (ES) = 0.75), while physical training interventions improved serve speed (ES = 0.72) and serve accuracy (ES = 1.14) among female tennis players. Additionally, Colomar et al. (2023) emphasized the importance of S&C practices (e.g., power-based and resistance training) in increasing tennis serve speed in their narrative review.

The complex bio-energetic demands of tennis make designing targeted S&C programs a challenging task for professionals (Reid and Schneiker, 2008). In the literature, individual studies assessing the effect of S&C training programs on tennis serve speed have shown inconsistent results. For example, Behringer et al. (2013) reported no significant improvement in serve speed among young tennis players following resistance training, in contrast to Baiget et al. (2023b), who observed beneficial effects. Similarly, while Egesoy et al. (2021) suggested that core training could increase serve speed, McCurdy et al. (2024) found that core training alone might not be effective. Although some types of S&C interventions have been shown to enhance serve speed, clear recommendations regarding the most effective or suitable interventions for tennis players are still lacking. A systematic review of the existing literature could provide valuable insights into the effectiveness of strength and conditioning training on serve speed in tennis players (Siddaway et al., 2019). Furthermore, a meta-analysis could consolidate data from diverse studies, offering a comprehensive evaluation of different training methods while uncovering patterns or resolving inconsistencies in individual study results. Accordingly, given the inconsistencies in the literature regarding the effects of S&C training on tennis serve speed, along with the limited number of systematic reviews and meta-analyses in this area, this study has two primary objectives: to update the existing body of knowledge and to identify interventions that effectively enhance serve speed in tennis players.

## Methodology

The present review was reported following the updated PRISMA statement (Page et al., 2021), and the review protocol was registered in PROSPERO (identifier CRD42024519790).

### Literature search

A literature search was conducted according to PRISMA in Web of Science, PubMed, SportsDiscuss, and Scopus on 19 July 2024. The following keywords and phrases were combined with Boolean operators: “strength” OR “conditioning” OR “resistance” OR “plyometric” OR “exercise*” AND “intervention*” OR “training” OR “program*” AND “serv* speed” OR “serv* velocity” AND “tennis.” We did not restrict the search by study date. Additionally, our review team conducted a thorough manual search of Google Scholar and the reference lists of all selected papers to ensure comprehensive coverage. Detailed search strings for each database are provided in the Appendix of the Supplementary Material.

### Eligibility criteria

The eligibility criteria for this review were established based on the PICOS (Population, Intervention, Comparator, Outcomes, and Study Design) framework.

Studies were considered eligible for inclusion in our review if they met the following criteria:

a. The studies recruited healthy tennis players (healthy tennis players refer to individuals who actively play tennis without injuries or medical conditions affecting performance or training) as subjects, with no restrictions on age, gender, or playing level.
b. The studies involved S&C training interventions (definitions provided in Table 1).
c. The outcome measure was related to serve speed.
d. The articles were randomized controlled trials (RCTs) and published in English.
e. The articles provided the mean and standard deviation of serve speed for both pre-test and post-test measurements.

**TABLE 1:** Definitions of training modes used for review.

Training mode	Definition
Resistance training	Resistance training refers to a form of exercise that uses external resistance, such as weights, resistance bands, or body weight. It includes various exercises like back squats, leg presses, lunges, calf raises, and deadlifts
Core training	Core training involves exercises and routines aimed at strengthening the core muscles, including those in the abdomen, lower back, hips, and pelvis
Multimodal training	This category encompasses experimental groups that implemented a combination of at least two of strength and conditioning training
Plyometric training	Plyometric training involves exercises that utilize the stretch-shortening cycle, emphasizing a quick shift from eccentric to concentric muscle contractions to generate explosive, high-speed movements like jumps or bounds

Studies were excluded based on the following criteria:

a. The studies were non-RCTs or secondary research (e.g., reviews).
b. Papers not related to tennis.
c. The studies involved unhealthy individuals (e.g., ankle sprain).
d. The studies did not provide adequate results.
e. The studies tested the effects of S&C training interventions without a control group.
f. The outcome measure was not related to tennis serve speed.
g. Training interventions did not include S&C training or combined S&C training with other physiological training methods (e.g., motor imagery).

### Methodological quality

Our review team assessed the methodological quality of each study included in the analysis using the PEDro scale (Maher et al., 2003). The PEDro scale includes 11 criteria designed to assess methodological quality. Each criterion met contributes one point to the total PEDro score, ranging from 0 to 10 points. For this analysis, criterion 1, which pertains to external validity, was excluded from the quality assessment. Studies were classified based on their PEDro scores as follows: high (6–10), moderate (4–5), or poor (≤3). Two reviewers (ND and XY) independently evaluated the studies, and Cohen’s kappa was used to quantify the level of agreement between their assessments. Any discrepancies were resolved through a voting process that included a third reviewer (KGS).

### Data extraction

Data from each study were extracted into a Microsoft Excel sheet (Microsoft Corporation, Redmond, WA, United States) and included the following details: author’s name, publication year, participant information (e.g., sample size, age, sex, and competition level), S&C training interventions (e.g., type, duration, and frequency), and outcome measures (e.g., serve speed). In addition, we extracted pre- and post-intervention test means and standard deviation data for the outcome measures in each study. Two authors (ND and XY) independently conducted the data extraction and the final sheet was cross-verified with the third author (KGS) to ensure completeness and accuracy.

### Statistical analyses

All extracted meta-analysis data were imported into the comprehensive meta-analysis software (Version 3.0; Biostat, Englewood, NJ, United States) for analysis and processing. Of note, meta-analyses were performed when at least two studies were available for each training intervention. The ES (i.e., Hedges’g) is a standardized measure that evaluates the extent of differences between groups or experimental conditions. In line with recommendations for sports science research, ES values were reported with 95% confidence intervals (CIs) and interpreted as follows: <0.2 (trivial), 0.2–0.6 (small), >0.6–1.2 (moderate), >1.2–2.0 (large), >2.0–4.0 (very large), and >4.0 (extremely large) (Hopkins et al., 2009). To standardize the results, post-intervention standard deviation values were utilized, and a random-effects model was applied to accommodate the variability among trials that could affect the outcomes of S&C training (Kontopantelis et al., 2013). In trials with multiple S&C training groups, the control group’s sample size was proportionately divided to ensure all subjects were included in comparisons (Higgins et al., 2008). When numerical data were not available in tables or supplementary materials but were presented in figures, we used Graph Digitizer software (Digitizelt, Germany) to extract the necessary data (mean and standard deviation) from the graphs (Drevon et al., 2016). Heterogeneity across studies was assessed using Q and I^2^ statistics. The I^2^ statistic was interpreted as follows: <25% indicates low heterogeneity, 25%–75% moderate heterogeneity, and >75% high heterogeneity (Higgins and Thompson, 2002). To evaluate the risk of publication bias, we employed the extended Egger’s test (Egger et al., 1997). When Egger’s test indicated significant bias, we applied a trimming and filling method. Additionally, to evaluate the robustness of our findings, we conducted sensitivity analyses using the one-study-removed approach. Statistical significance was determined using a threshold of *p* < 0.05.

## Results

### Study selection

**FIGURE 1:**
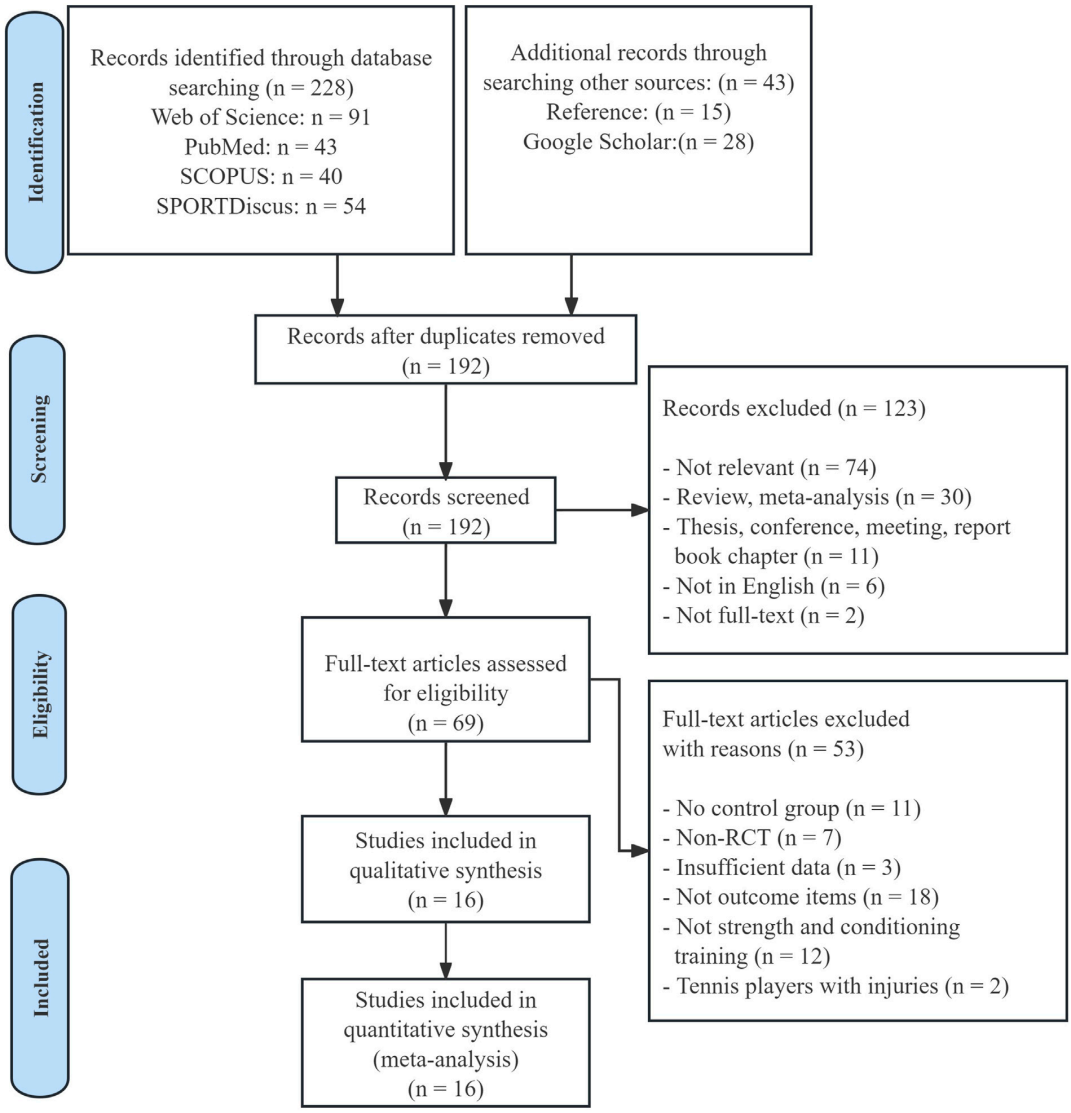
Flow diagram of the study selection.

A total of 228 potential studies relevant to the research topic were identified through database searches, supplemented by 28 articles from Google Scholar and 15 additional studies obtained from reference lists. After removing duplicates, we screened the titles and abstracts of the remaining studies, resulting in 69 full-text articles that warranted further evaluation. These articles were then meticulously assessed based on predefined inclusion and exclusion criteria to determine their suitability for the study. Ultimately, 16 RCTs met all the inclusion criteria and provided sufficient data to be included in the meta-analysis, as illustrated in Figure 1.

### Methodological quality

Table 2 presents the methodological quality ratings of all selected studies according to the PEDro scale. Of the sixteen RCTs, nine recorded a PEDro score of 6 or 7, signifying high quality, while the remaining seven scored 4 or 5, indicating moderate quality.

**TABLE 2:** Physiotherapy Evidence Database (PEDro) scale ratings.

Study name	Eligibility criteria	Random allocation	Concealed allocation	Group similar at baseline	Blind subject	Blind therapist	Blind assessor	Follow-up	Intention- to-treat analysis	Between-group comparisons	Point measure and variability	Total*	Study quality
Mont et al. (1994)	1	1	0	1	0	0	0	1	0	1	1	5	Moderate
Treiber et al. (1998)	1	1	0	1	0	0	0	1	1	1	1	6	High
Kraemer et al. (2000)	0	1	0	1	0	0	0	1	1	1	1	6	High
Kraemer et al. (2003)	1	1	0	1	0	0	0	1	1	1	1	6	High
Malliou et al. (2011)	0	1	0	1	0	0	0	1	0	0	1	4	Moderate
Behringer et al. (2013)	1	1	1	1	0	0	0	1	1	1	1	7	High
Terraza-Rebollo et al. (2017)	1	1	0	1	0	0	0	1	0	0	1	4	Moderate
Canós et al. (2022a)	1	1	0	1	0	0	0	1	1	1	1	6	High
Canós et al. (2022b)	1	1	1	1	0	0	0	1	1	1	1	7	High
Baiget et al. (2023b)	1	1	0	1	0	0	0	1	1	1	1	6	High
McCurdy et al. (2024)	0	1	0	1	0	0	0	1	0	0	1	4	Moderate
Egesoy et al. (2021)	1	1	0	1	0	0	0	1	1	1	1	6	High
Liu, 2022	0	1	0	1	0	0	0	1	0	1	1	5	Moderate
Fernandez-Fernandez et al. (2013)	1	1	0	1	0	0	0	1	0	1	1	5	Moderate
Kara et al. (2015)	0	1	0	1	0	0	0	1	0	1	1	5	Moderate
Fernandez-Fernandez et al. (2020)	1	1	0	1	0	0	0	1	1	1	1	6	High

Notes: A detailed explanation for each PEDro scale item can be accessed at https://www.pedro.org.au/english/downloads/pedro-scale.

*From a possible maximal punctuation of 10.

### Categories of intervention summaries

The 16 studies were categorized into four intervention types: resistance training (n = 10), core training (n = 3), multimodal training (n = 3), and plyometric training (n = 1). Table 3 summarized the general characteristics of all the individual trials selected in this systematic review and meta-analysis.

**TABLE 3:** Characteristics of included studies.

Study	Participants				Training program						Outcome
	n	Age (year)	Gender	Level	Intervention	Weeks	Freq	Sets x reps	RBS (s)	Intensity	SP
Resistance training											
Mont et al. (1994)	30	18–42	M	Elite	EG1: isokinetic concentric training (n = 8); EG2: isokinetic eccentric training (n = 9); CG: no training (n = 13)	6	3	8 × 10	60	NR	EG1: 11%↑ EG2: 11%↑
Treiber et al. (1998)	22	18–29	Mixed	Collegiate	EG: shoulder resistance training (n = 11); CG: regular training (n = 11)	4	3	2 × 20	30–40	NR	6%↑
Kraemer et al. (2000)	24	EG1:19.0 ± 0.9 EG2:18.9 ± 1.2 CG:19.8 ± 1.7	F	Collegiate	EG1: periodized resistance training (n = 8); EG2: single-set circuit resistance training (n = 8); CG: regular training (n = 8)	36	2–3	8–10	60–120	NR	EG1: 2.8%↑ EG2: 1.4%↑
Kraemer et al. (2003)	28	19 ± 1.0	F	Collegiate	EG1: periodized resistance training (n = 10); EG2: non-periodized training (n = 10); CG: regular training (n = 8)	36	2	2–3	90–120	4–15 RM	EG1: 29%↑ EG2: 16%↑
Malliou et al. (2011)	56	13–14	Mixed	Clubs	EG: shoulder resistance training (n = 27); CG: regular training (n = 29)	7	3	2–3×10-15	NR	NR	1.7%↑
Behringer et al. (2013)	23	15.03 ± 1.64	M	Clubs	EG: resistance Training (13) CG: regular training (n = 10)	8	2	2 × 15	60	65%–85% lRM	1.18%↑
Terraza-Rebollo et al. (2017)	20	15.5 ± 0.9	Mixed	NR	EG1: overloads resistant training (n = 7); EG2: resistant training with medicine ball throws and elastic bands (n = 7); CG: regular training (n = 6)	8	3	EG1: 3 × 1 EG2: 3 × 6	180	NR	EG1: 4%↑ EG2: 1%↑
Canós et al. (2022a)	24	EG1: 15.5 ± 1.2 EG2: 15.5 ± 1.2 CG: 15.9 ± 1.0	M	NR	EG1: machine-based resistant training (n = 8); EG2: flywheel-based resistance training (n = 8); CG: regular training (n = 8)	8	2	3×6–8	90	EG1: 6.2 ± 0.6 RPE EG2: 6.1 ± 0.7 RPE	EG1: 1.8%↑ EG2: 2.5%↑
Canós et al. (2022b)	24	EG1: 15.6 ± 1.0 EG2: 15.8 ± 0.7 CG: 15.6 ± 0.9	F	Club	EG1: machine-based resistant training (n = 7); EG2: flywheel-based resistance training (n = 8); CG: regular training (n = 9)	8	2	3×6–8	90	50%–70%1RM	EG1: 3.4%↑ EG2: 1.9%↑
Baiget et al. (2023b)	18	EG: 15.6 ± 1.1 CG: 15.5 ± 0.6	M	Elite	EG: isokinetic training (n = 10) CG: Usual physical and technical–tactical training (n = 6)	6	2–3	1×3–5	45	100%MVIC	7.0%↑
Core training											
McCurdy et al. (2024)	35	F = 23.43 ± 5.2 M = 27.95 ± 7.5	Mixed	University	EG: core training (n = 17) CG: no training (n = 18)	8	2	2–3×4–7	30–45		2%↑
Egesoy et al. (2021)	36	11.75 ± 0.5	Mixed	NR	EG1: static Core training (n = 12); EG2: dynamic core training (n = 12) CG: regular training (n = 12)	8	2	2 × 1	10–20	NR	EG1: 5.96%↑ EG2: 6.24%↑
Liu, 2022	20	NR	NR	Collegiate	EG: core training (n = 10); CG: regular training (n = 10)	14	3	NR	NR	NR	8.24%↑
Multimodal training											
Fernandez-Fernandez et al. (2013)	30	14.2 ± 0.5	M	National	EG: combined training (core strength, elastic resistance and medicine ball exercises) (n = 15) CG: regular training (n = 15)	6	3	2 × 8	60s	NR	5%↑
Kara et al. (2015)	20	EG: 22.8 ± 1.6 CG:18 ± 0.0	NR	NR	EG: combined training (medicine ball, resistance and balance exercises) (n = 10); CG: routine training	6	3	2–4×6–10	30–90	NR	23.57%↑
Fernandez- Fernandez et al. (2020)	29	15.09 ± 1.16	M	Well-trained	EG: combined training (general mobility, core, and shoulder strength exercises, plyometric and acceleration/deceleration/COD drills) (n = 14); CG: dynamic warm-up training (n = 15)	8	3	2–3×6–15	10	NR	7.7%↑
Plyometric training											
Behringer et al. (2013)	20	15.03 ± 1.64	M	Clubs	EG: plyometric Training (n = 10) CG: regular training (n = 10)	8	2	3–4×10-15	20	65%–85% l RM	3.78%↑

M, male; F, female; NR, not reported; Freq, frequency; EG, experimental group; PRE, modified Börg’s rate of perceived exertion; MVIC, maximal voluntary isometric contraction; reps, repetitions; RM: repetition maximum; COD, change of direction; RBS, rest between sets; SP, serve speed.

### Resistance training

This review identified ten studies (67%, n = 269) that investigated the effectiveness of resistance training on serve speed in tennis players (Table 3). Among these, three studies focused on females (Kraemer et al., 2000; Kraemer et al., 2003; Canós et al., 2022b), four on males (Mont et al., 1994; Behringer et al., 2013; Canós et al., 2022a; Baiget et al., 2023b), and the remaining three included both sexes (Treiber et al., 1998; Malliou et al., 2011; Terraza-Rebollo et al., 2017). Participants’ ages ranged from approximately 13–42 years. Three studies focused on collegiate-level players (Treiber et al., 1998; Kraemer et al., 2000; 2003), three recruited club-level players (Malliou et al., 2011; Behringer et al., 2013; Canós et al., 2022b), two targeted elite players (Mont et al., 1994; Baiget et al., 2023b), and two did not report this information (Terraza-Rebollo et al., 2017; Canós et al., 2022a). The resistance training methods included shoulder resistance training, periodized resistance training, single-set resistance training, circuit resistance training, machine-based resistance training, and flywheel-based resistance training. Participants trained 2 to 3 times per week for durations ranging from 4 to 36 weeks, totaling 8 to 72 sessions. Training volume varied from 2 to 8 sets per exercise and 1 to 20 repetitions.

### Core training

This review identified three studies (20%, n = 91) that evaluated the impact of core training on serve speed in tennis players (Table 3). Two of these studies involved participants of mixed genders, with ages ranging from approximately 11–28 years (McCurdy et al., 2024; Egesoy et al., 2021). Two studies specified that the tennis players recruited competed at the collegiate/university level (McCurdy et al., 2024; Egesoy et al., 2021), while the third study did not provide this information (Liu, 2022). Core training types included static and/or dynamic exercises. Participants trained 2 to 3 times per week for 8–14 weeks, totaling 16 to 42 sessions. Training volume ranged from 2 to 3 sets per exercise and 1 to 7 repetitions.

### Multimodal training

This review identified three studies (20%, n = 79) that evaluated the impact of multimodal training on serve speed in tennis players (Table 3). Two studies focused on male participants (Fernandez-Fernandez et al., 2013; 2020), while the third did not report gender (Kara et al., 2015). Participants’ ages ranged from 14 to 23 years. One study involved well-trained tennis players (Fernandez-Fernandez et al., 2020), another recruited national-level tennis players (Fernandez-Fernandez et al., 2013), and the third did not specify player levels (Kara et al., 2015). Multimodal training included core strength, elastic resistance, medicine ball exercises, balance, shoulder strength exercises, and plyometric and acceleration/deceleration/change of direction drills. Participants trained 3 times per week for 6–8 weeks, totaling 8 to 36 sessions, with a volume of 2–4 sets per exercise and 6 to 15 repetitions.

### Plyometric training

Due to the inclusion of only one study (Behringer et al., 2013) involving plyometric training, this type of training was excluded from the meta-analysis. In Behringer et al.'s study, thirty-six male tennis players (mean age 15.03 ± 1.64 years) were randomly allocated to groups. The experimental group engaged in plyometric exercises twice weekly for 8 weeks, performing activities such as skipping, hopping, jumping, push-ups, and medicine ball exercises. The regimen involved 3 to 4 sets per exercise, with 10–15 repetitions per set. Compared to the control group, the plyometric group demonstrated a significant increase in serving speed, improving by 3.8 ± 4.5 kph (Behringer et al., 2013).

### Meta-analysis results

**FIGURE 2:**
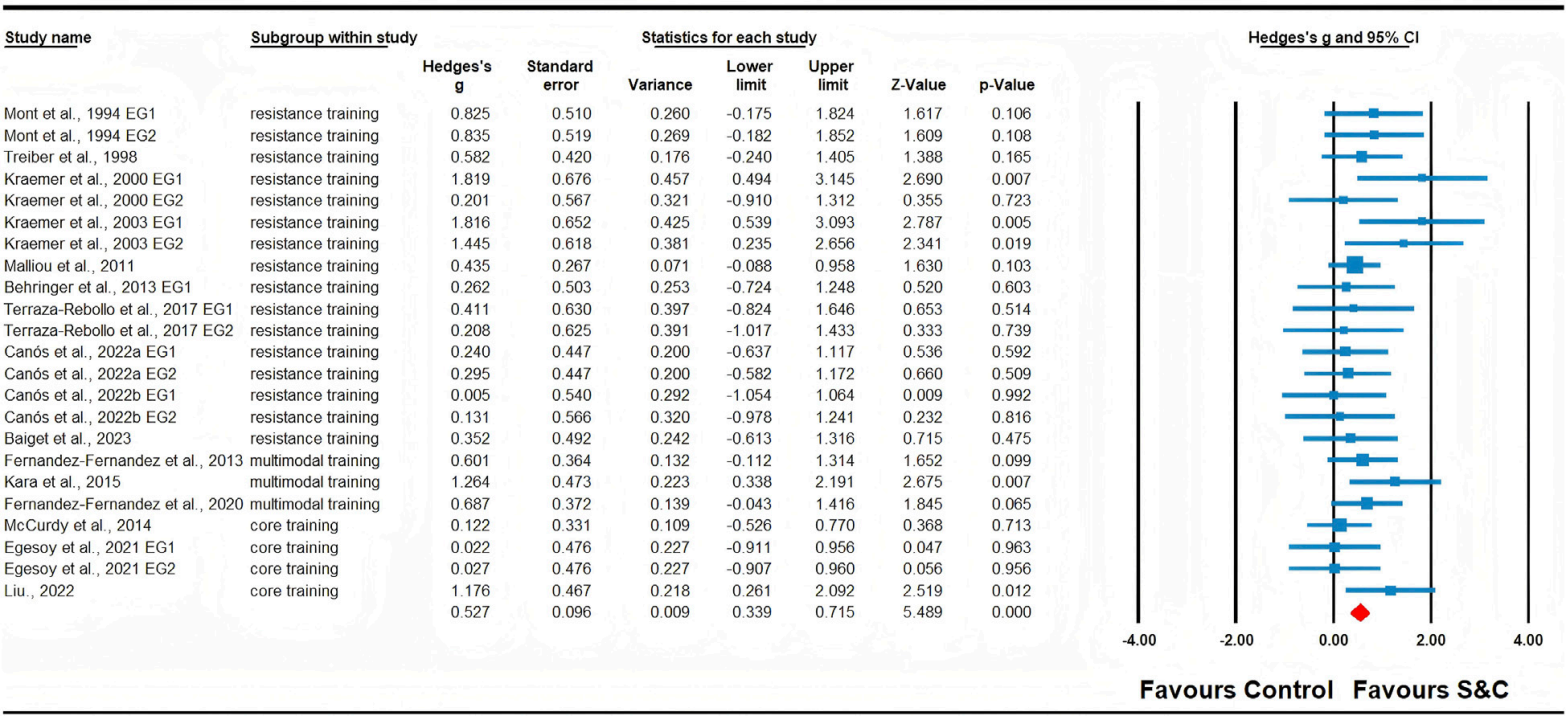
Forest plot of changes in serve speed in tennis players participating in strength and conditioning (S&C) training compared to controls. Values shown are effect sizes (Hedges’s g) with 95% confidence intervals (CI). The size of the plotted squares reflects the statistical weight of the study. EG: experimental group.

**FIGURE 3:**
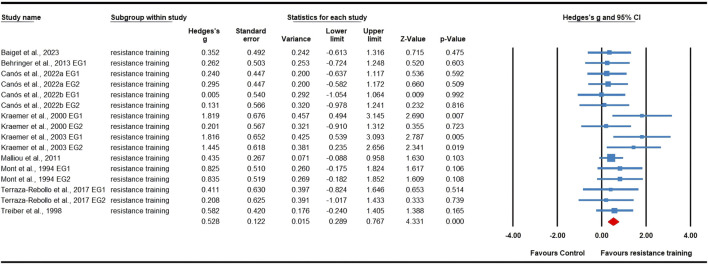
Forest plot of changes in serve speed in tennis players participating in resistance training compared to controls. Values shown are effect sizes (Hedges’s g) with 95% confidence intervals (CI). The size of the plotted squares reflects the statistical weight of the study. EG: experimental group.

**FIGURE 4:**
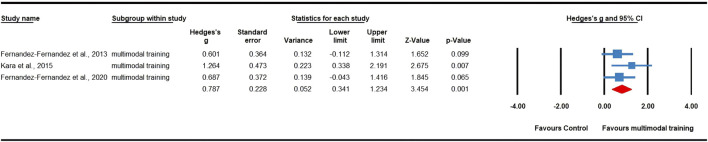
Forest plot of changes in serve speed in tennis players participating in multimodal trainingcompared to controls. Values shown are effect sizes (Hedges’s g) with 95% confidence intervals (CI). The size of the plotted squares reflects the statistical weight of the study. EG: experimental group.

**FIGURE 5:**
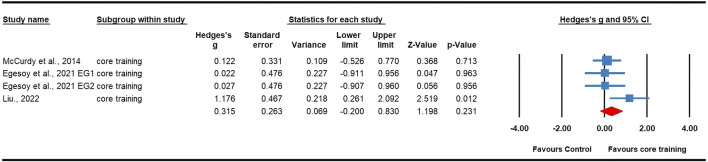
Forest plot of changes in serve speed in tennis players participating in core training compared to controls. Values shown are effect sizes (Hedges’s g) with 95% confidence intervals (CI). The size of the plotted squares reflects the statistical weight of the study. EG: experimental group.

Twenty-three effects were analyzed from 16 original RCTs. Overall, S&C training was associated with a significant increase in tennis serve speed (ES = 0.53; 95% CI = 0.34 to 0.72; *p* < 0.0001; Figure 2). Subgroup analyses showed significant improvements in serve speed after resistance training (ES = 0.53, small; 95% CI = 0.29 to 0.77; *p* < 0.0001; I^2^ = 0.00%; Figure 3) and multimodal training (ES = 0.79, moderate; 95% CI = 0.34 to 1.23; *p* = 0.001; Figure 4), with no evidence of heterogeneity across studies for either subgroup (I^2^ = 0.00%, Q = 13.73, *p* = 0.546; and I^2^ = 0.00%, Q = 1.35, *p* = 0.508, respectively). There was no significant change detected following core training (ES = 0.32; 95% CI = −0.20 to 0.83; *p* = 0.231; Figure 5), with a low level of heterogeneity observed (I^2^ = 33%; Q = 0.49; *p* = 0.213). In addition, no significant differences among subgroups were noted (*p* = 0.289).

### Assessment of publication bias and sensitivity analyses

To assess potential publication bias in our meta-analysis, we used Egger’s linear regression test. The Egger’s test results indicated no significant publication bias for resistance training (regression intercept = 1.26, *p* = 0.183) or core training (regression intercept = 1.79, *p* = 0.719). Conversely, significant publication bias was found for multimodal training (regression intercept = 1.79, *p* = 0.036). Nonetheless, after adjusting for publication bias with the trim-and-fill method, the overall ESs remained consistent. In the sensitivity analysis, where each trial was individually excluded from the model, the results remained consistent.

## Discussion

This systematic review and meta-analysis aimed to evaluate the impact of S&C training on serve speed in tennis players. Sixteen RCTs met the inclusion criteria for the meta-analysis. The main findings indicated that S&C training significantly increased serve speed. Additionally, both resistance training and multimodal training interventions were found to be effective strategies for enhancing serve speed.

### The effect of resistance training on serve speed

The present meta-analysis indicated that resistance training was effective in enhancing serve speed (ES = 0.53) in tennis players. This form of training has been demonstrated to improve muscle power, strength, and hypertrophy in a variety of athletes, making it an integral aspect of athletic performance (Naclerio et al., 2013; Granacher et al., 2016; Dhahbi et al., 2024a). Athletes can develop more forceful movements by increasing muscle mass and force production capabilities, which directly translate into enhanced performance indicators such as serve speed (Deng et al., 2022). Deng et al. (2023) demonstrated that physical training (i.e., resistance training) positively influenced serve speed in female tennis players (ES = 0.72). Furthermore, multiple studies have demonstrated that resistance training improves ball speed in overhead athletes (Van den Tillaar, 2004; Van den Tillaar and Marques, 2009; Oranchuk et al., 2021; Job et al., 2022). Notably, the majority of resistance training programs included in our analysis were primarily aimed at enhancing strength in the shoulder joint (Mont et al., 1994; Treiber et al., 1998; Malliou et al., 2011; Baiget et al., 2023b). Baiget et al. (2016) found a significant relationship between the maximal isometric strength of shoulder internal rotation and serve speed, suggesting that maximal isometric strength in shoulder flexion and internal rotation are reliable predictors of ball speed during serves. The tennis serve requires substantial shoulder joint capabilities, particularly during racquet acceleration (Abrams et al., 2014). The force-time characteristics of the shoulder joint, such as the rate of force generation and impulse, are closely linked to serving speed in high-performance players (Baiget et al., 2016). With this in mind, resistance training had a significant positive impact on shoulder internal rotation and flexion, resulting in improved serve speed.

Several studies indicated a significant association between medicine ball throwing and functional performance (e.g., serving/throwing speed) (Raeder et al., 2015; Taniyama et al., 2021; Terraza-Rebollo and Baiget, 2021). Nevertheless, one study in our review (Canós et al., 2022a) found that although medicine ball throwing performance improved with machine- and flywheel-based resistance training, this did not lead to increased serve speed, indicating that these measures may not be strongly correlated. The authors indicate that brief periods of specific practice in throwing medicine balls may be insufficient to enhance serve speed (Canós et al., 2022a). Similarly, a study conducted by Canós et al. (2022b) discovered significant enhancements in serve speed between the fourth and eighth weeks for the group that underwent machine resistance training. However, no increases were seen in medicine ball throwing. In contrast, the group that received flywheel resistance training demonstrated improvement in all measures of throwing performance, except for an increase in serve speed. Moreover, the consensus among studies is that the tennis serve is a multifaceted motor skill that is affected by many physical parameters, including strength, range of motion, and technique (Fett et al., 2020; Colomar et al., 2022). Hence, it is essential for tennis players to participate in training programs that accurately replicate actions, with a focus on efficiently transmitting force along the full power chain, including trunk core stability and pelvic rotation (Colomar et al., 2023). Reid and Schneiker (2008) suggested incorporating resistance training tailored to the specific demands of tennis, such as enhancing the strength of rotator cuff muscles, into regular or supplementary training sessions. Overall, resistance training can offer benefits, especially when it comes to improving the speed of a tennis player’s serve.

### The effect of core training on serve speed

The analysis revealed that core training did not significantly change serve speed (ES = 0.32). In contrast, studies consistently demonstrated the effectiveness of core training approaches in enhancing ball speed in players. For instance, Saeterbakken et al. (2011) implemented core training regimens with handball players. Following a 6-week training session, there was an observed improvement in throwing speed. Prokopy et al. (2008) used core training and saw an improvement in throwing speed among softball players. A previous meta-analysis conducted by Lin et al. (2023) reported that core training led to a substantial increase in standing ball throwing speed among overhead-throwing athletes. Rodríguez-Perea et al. (2023) reported that core training enhanced both the speed (ES = 0.30) and distance (ES = 3.42) of throwing/hitting actions. The improvement in throwing speed was attributed to strengthened core muscles, which enhanced kinetic chain efficiency by stabilizing the spine and pelvis and enabling effective energy transfer between the upper and lower body (Manchado et al., 2017; Chu et al., 2016).

Core training that targets local muscles for posture control enhances the stability required for efficient strength and power production from global muscles during high-intensity, sport-specific movements (Hibbs et al., 2008). However, despite the time-saving aspect of core-only training, it is not effective in increasing serve speed in tennis players (McCurdy et al., 2024). The speed of a tennis serve is affected by the transfer of angular momentum through the kinetic chain, involving force generation from the legs, through the core, and into the upper body (Abrams et al., 2014). While core training enhances core strength, stability, and control, it may not necessarily contribute directly to the explosive power required for increasing serve speed. Other factors, such as shoulder strength and technique, play a significant role in determining serve speed (Kovacs and Ellenbecker, 2011; Baiget et al., 2016). These factors may explain the lack of significant gains in serve speed observed in our meta-analysis following core training interventions.

It is important to note that training effects are often influenced by the training protocol, and studies reporting on the effects of different core training programs on core muscle function and ball speed may have varying results. According to a systematic review by Oyama and Palmer (2023), more challenging core exercises that involve higher resistance, unstable surfaces, and dynamic trunk movements are necessary to improve ball-throwing speed. Similarly, Saeterbakken et al. (2011) propose that core training with unstable, closed kinetic chain exercises can substantially improve maximal throwing speed in handball players. Furthermore, elite tennis players have mastered neuromuscular patterns and techniques to a level that results in high racket speed (Kovacs and Ellenbecker, 2011). Thus, improving serve speed becomes increasingly challenging as playing ability advances. Dhahbi et al. (2024b) highlight that improving the understanding of load quantification across various training modalities could enhance training prescriptions and provide deeper insights into dose–response relationships within different approaches. Moreover, a significant association has been identified between aerobic fitness levels and the recovery capacity of athletes following high-intensity, short-duration efforts (Campos et al., 2012; Dhahbi et al., 2024c). Accordingly, further empirical investigations are warranted to elucidate the relationship between core training and serve speed.

### The effect of multimodal training on serve speed

Based on the findings of this meta-analysis, incorporating multimodal training into interventions might have been beneficial, as it was shown to enhance serve speed in tennis players (ES = 0.79). The performance enhancements observed in this study align with findings from other research on multimodal training and ball speed (Van den Tillaar, 2004; Dahl and van den Tillaar, 2021; Le Solliec et al., 2023). Fernandez-Fernandez et al. (2013), Fernandez-Fernandez et al. (2020) investigated the effects of 6–8 weeks of multimodal training in well-trained tennis players, incorporating whole-body exercises, and found positive results in serve speed. A 12-week multimodal training program targeting the major muscle groups of the core, upper body, and lower body significantly improved ball-shooting speed in young soccer players (Wong et al., 2010). A meta-analysis by Deng et al. (2023) revealed that physical training significantly improved serve speed in female tennis players (ES = 0.72). Biomechanical analysis of the tennis serve indicates that kinetic energy is generated almost equally by the upper and lower extremities throughout the motion (Martin et al., 2014). Furthermore, an electromagnetic investigation reported that muscle activation during overarm throwing progresses gradually from the trunk to the arm (Hirashima et al., 2002), reinforcing the importance of integrated movement patterns for improving ball speed (Myers et al., 2015). Therefore, multimodal training interventions may be an effective method to improve tennis players’ serve speed, as experts have demonstrated that these athletes typically use the entire kinetic chain, integrating multiple anatomical segments and regions to generate force in a proximal-to-distal manner (Colomar et al., 2022; Ličen et al., 2022).

### The effect of plyometric training on serve speed

There is scarce research on the effects of plyometric training on tennis players, with only one RCT examining its impact on serve speed (Behringer et al., 2013). This study found that an 8-week plyometric training program significantly increased serve speed compared to regular tennis training. Systematic reviews and meta-analyses have previously demonstrated the benefits of plyometric training in overhead athletes (Myers et al., 2015; Eraslan et al., 2021). Notably, Deng et al. (2022) reported a significant positive effect of plyometric training programs on maximal serve speed (ES = 0.75). Moreover, their meta-analysis on the effects of plyometric training on technical skills in athletes highlighted improvements in throwing speed (ES = 0.37–0.78) across sports such as handball, baseball, and water polo (Deng et al., 2023). Additionally, several studies have demonstrated the effectiveness of plyometric training in enhancing kicking speed (5%–14%) among soccer players (Campo et al., 2009; Sedano et al., 2011; Ramírez-Campillo et al., 2015). It is suggested that plyometric training enhances intermuscular coordination, thereby improving force transfer through the kinetic chain (Fernandez-Fernandez et al., 2016). Research has also identified a connection between strength, power, and ball speed in tennis players (Baiget et al., 2016; Hayes et al., 2021). Plyometric training is widely recognized as an effective method to enhance strength and power (Makaruk and Sacewicz, 2010; De Villarreal et al., 2010; Stojanović et al., 2017). Despite its potential benefits, few studies have specifically examined the impact of plyometric training on tennis serve speed. Therefore, further research is necessary.

## Limitations

To our knowledge, this is the first meta-analysis to comprehensively and quantitatively assess the impact of S&C training on serve speed among tennis players. Despite its novelty, this meta-analysis has several limitations. Firstly, we reported only one outcome measure (i.e., serve speed) for comparison across S&C training interventions. Serve speed is the most widely used performance measure in tennis and aligns with the purpose of this systematic review. Secondly, the limited number of studies for each training method prevents the identification of the most effective dose-response relationship between S&C training and serve speed. Additionally, the analysis of moderating variables such as age, gender, and competitive level is limited. The considerable heterogeneity in training protocols and other serve speed-related factors (e.g., practice and competition) across studies makes it challenging to identify the appropriate training parameters. Thirdly, some included studies lacked clear descriptions of their training protocols, such as the number of sets and repetitions, intensity, session duration, and rest between sets. Finally, there was only one RCT on plyometric training, preventing a meta-analysis from being performed. Finally, this review included only studies published in English, as it is the most widely used language in research. However, some studies relevant to this systematic review may exist in other languages, highlighting the need for future researchers to incorporate studies published in non-English languages.

### Practical applications

The findings from our work are valuable for S&C coaches and practitioners in designing and implementing tennis-specific training programs to improve serve speed. Overall, the data from this meta-analysis indicated that increasing serve speed in tennis players could be achieved through S&C training. The results showed that multimodal training interventions lasting 6–8 weeks, with 3 sessions per week, 2–8 sets, and 10–15 repetitions, were effective for enhancing serve speed. Similarly, resistance training programs spanning 4–36 weeks, with 2–3 sessions per week, 1–8 sets, and 3–10 repetitions, demonstrated positive effects on serve speed. In contrast, core training did not demonstrate an improvement in tennis serve speed. We recommend future trials compare the effects of various S&C programs or parameters within the same program to identify the most beneficial conditions.

## Conclusion

The findings indicated that S&C interventions, such as resistance and multimodal training, effectively enhanced serve speed in tennis players. Among these, the training program that yielded the largest ES on serve speed utilized multimodal exercises targeting both upper and lower extremities. However, core training did not show positive effects. Further studies on tennis players’ serve speed are needed to confirm or refute this conclusion.

## Data Availability

The original contributions presented in the study are included in the article/Supplementary Material, further inquiries can be directed to the corresponding author.
