# Supplementary material for: The effects of strength and conditioning interventions on serve speed in tennis players: a systematic review and meta-analysis
Source: Front Physiol. 2025 Jan 7;15:1469965. doi: 10.3389/fphys.2024.1469965 (PMC11747802; doi:10.3389/fphys.2024.1469965)
Supplement: Supplementary file 1 [file Table1.docx]

**Detailed search strategy**

**Search on 19 July 2024**

| **Databases** | **search strategy** | **Results** |
| --- | --- | --- |
| PubMed | (("strength"[Title/Abstract] OR "conditioning"[Title/Abstract] OR "resistance"[Title/Abstract] OR "plyometric"[Title/Abstract] OR "exercise*"[Title/Abstract] AND "intervention*"[Title/Abstract] OR "training"[Title/Abstract] OR program*[Title/Abstract]) AND ("serv* speed"[Title/Abstract] OR "serv* velocity"[Title/Abstract])) AND ("tennis"[Title/Abstract]) | 43 |
| Web of Science | ((AB=( “strength” OR “conditioning” OR “resistance” OR “plyometric” OR “exercise*” AND “intervention*” OR “training” OR “program*”)) AND AB=(“serv* speed” OR “serv* velocity”)) AND AB=(“tennis”) | 91 |
| SPORTDicus | AB (“strength” OR “conditioning” OR “resistance” OR “plyometric” OR exercise*” AND “intervention*” OR “training” OR “program*”) AND AB (“serv* speed” OR “serv* velocity”) AND AB “tennis” | 54 |
| Scopus | ( TITLE-ABS-KEY ( "strength" OR "conditioning" OR "resistance" OR "allometric" OR "exercise*" AND "intervention*" OR "training" OR "program*" ) AND TITLE-ABS-KEY ( "serv* speed" OR "serv* velocity" ) AND TITLE-ABS-KEY ( "tennis" ) ) | 40 |
| Total |  | 228 |
